# Eating and drinking interventions for people at risk of lacking decision-making capacity: who decides and how?

**DOI:** 10.1186/s12910-015-0034-8

**Published:** 2015-06-11

**Authors:** Gemma Clarke, Sarah Galbraith, Jeremy Woodward, Anthony Holland, Stephen Barclay

**Affiliations:** Palliative and End of Life Care Group, Department of Public Health and Primary Care, Institute of Public Health Forvie Site, University of Cambridge School of Clinical Medicine, Box 113 Cambridge Biomedical Campus, CB2 0SR Cambridge, UK; Department of Palliative Care, Box 63, Addenbrooke’s Hospital, Hills Road, CB2 0QQ Cambridge, UK; Department of Gastroenterology, Box 133, Addenbrooke’s Hospital, Hills Road, CB2 0QQ Cambridge, UK; Cambridge Intellectual & Developmental Disabilities Research Group, Department of Psychiatry, University of Cambridge, Douglas House, 18B Trumpington Road, CB2 8AH Cambridge, UK

**Keywords:** Decision-making capacity, Healthcare decisions, Multi-professional, Artificial nutrition, Eating and drinking interventions, Progressive neurological disease

## Abstract

**Background:**

Some people with progressive neurological diseases find they need additional support with eating and drinking at mealtimes, and may require artificial nutrition and hydration. Decisions concerning artificial nutrition and hydration at the end of life are ethically complex, particularly if the individual lacks decision-making capacity. Decisions may concern issues of life and death: weighing the potential for increasing morbidity and prolonging suffering, with potentially shortening life. When individuals lack decision-making capacity, the standard processes of obtaining informed consent for medical interventions are disrupted. Increasingly multi-professional groups are being utilised to make difficult ethical decisions within healthcare. This paper reports upon a service evaluation which examined decision-making within a UK hospital Feeding Issues Multi-Professional Team.

**Methods:**

A three month observation of a hospital-based multi-professional team concerning feeding issues, and a one year examination of their records. The key research questions are: a) How are decisions made concerning artificial nutrition for individuals at risk of lacking decision-making capacity? b) What are the key decision-making factors that are balanced? c) Who is involved in the decision-making process?

**Results:**

Decision-making was not a singular decision, but rather involved many different steps. Discussions involving relatives and other clinicians, often took place outside of meetings. Topics of discussion varied but the outcome relied upon balancing the information along four interdependent axes: (1) Risks, burdens and benefits; (2) Treatment goals; (3) Normative ethical values; (4) Interested parties.

**Conclusions:**

Decision-making was a dynamic ongoing process with many people involved. The multiple points of decision-making, and the number of people involved with the decision-making process, mean the question of ‘who decides’ cannot be fully answered. There is a potential for anonymity of multiple decision-makers to arise. Decisions in real world clinical practice may not fit precisely into a model of decision-making. The findings from this service evaluation illustrate that within multi-professional team decision-making; decisions may contain elements of both substituted and supported decision-making, and may be better represented as existing upon a continuum.

## Background

### Decision-making capacity

The concept of ‘decision-making capacity’ refers to a person’s ability to understand, retain and balance information, and communicate a choice [1]. It is a psychological construct which has been enshrined in law within some countries, for example; in England and Wales,^1^ and in Scotland.^2^ Decision-making capacity can be affected by many factors, such as: intoxication from the consumption of alcohol, an acquired injury to the brain, the presence of a developmental disability associated with cognitive impairment, or the presence of a brain disorder such as dementia. Loss of capacity can be temporary, fluctuating or permanent.

Over the past ten years the concept of decision-making capacity has grown in global significance, both within medicine, and across law, research, social care and human rights [2–4]. The number of individuals who may have a disorder which puts them at risk of lacking decision-making capacity is increasing as the age of the global population rises [5]. Research undertaken by Alzheimer’s Disease International predicts that the current 36 million people living with dementia will increase to 115 million by 2050 [6]. Advances in healthcare have also resulted in increased life expectancy for those with severe intellectual disabilities, [7] and those with acquired brain injuries such as stroke, [8–10] many of whom will have impairments in cognition and communication. Within high resource countries, improvements in medical treatments have resulted in the increased survival of very low birth weight babies, a proportion of whom will have significant physical and cognitive impairments in childhood and later life [11, 12].

The United Nations Convention on the Rights of Persons with Disabilities (UNCRPD) (2006)^3^ requires signatory parties to protect and promote the full human rights of persons with disabilities, including those at risk of lacking decision-making capacity [13]. The UNCRPD views disability through the lens of human rights, Article 12 guarantees that persons with disabilities, “enjoy legal capacity on an equal basis with others in all aspects of life” (UNCRPD, Article 12 (2)) [13]. This means that countries must “provide access by persons with disabilities to the support they may require in exercising their legal capacity” (UNCRPD, Article 12 (3)) [13].

### Decision-making in national and international law

Within one jurisdiction, England and Wales, the Mental Capacity Act 2005 (MCA) provides a statutory framework for how to proceed when people are judged not to have the mental capacity to make a particular decision. A person is only considered to be lacking decision-making capacity for a particular decision if: all practical steps have been taken without success, they have a disorder or impairment of the brain or mind, and are unable to: understand the relevant information; retain the information; weigh the information; communicate their decision [14]. Within Scotland, decision-making is covered by the Adults With Incapacity (Scotland) Act (2000) [15]. Similarly, in most US jurisdictions, individuals are expected to be able to demonstrate four abilities to show decision-making capacity: appreciate the nature and consequences of their own situation; understand the relevant information; be able to reason about potential risks and benefits; and communicate a choice [16].

The Mental Capacity Act 2005 requires that any decision made for an individual is in accordance with their ‘best interests’. Best interests are defined broadly, including: not making assumptions on the basis of age, appearance, condition or behaviour [14]. Advance decisions to refuse treatment (previously known as living wills), must be followed if valid and applicable, even if it may result in the person’s death [17]. Expressions of treatment preferences, or ‘advance expressions of preferences’ are not legally binding but should be incorporated as part of the decision-making process when assessing “best interests” [17].

Some critics have argued that assessments of capacity within the Mental Capacity Act 2005 may not be compliant with the United Nations Convention on the Rights of Persons with Disabilities (UNCRPD) [18, 19]. A strong reading of Article 12 of the UNCRPD, rejects the very concept of decision-making incompetence, arguing that there is no point at which legal capacity should be considered to be lost [18]. Such approaches push the focus away from capacity testing and substituted decision-making, towards an evaluative approach to decision-making abilities, in which a person’s support requirements for decision-making are determined and then met [20].

### Feeding issues at the end of life

Mealtimes are an important part of everyday life [21, 22]. The sharing of food represents, not only a way to sustain life, but also presents opportunities for pleasure, comfort, socialising and maintaining family and cultural identities [21, 23]. Individuals with certain conditions may find that they are unable to participate in mealtimes. For example, people with dementia, [24] intellectual disabilities [25] and acquired brain injuries, [26, 27] can find they are unable to take food and fluids orally, and/or may require additional support during mealtimes. When this occurs, decisions concerning artificial methods of nutrition and hydration may be required.

The term ‘artificial nutrition and hydration’ refers to interventions which allow people to receive nutrients and fluids through tubes directly into the digestive or venous system [28]. These interventions may also be referred to as ‘clinically-assisted nutrition’, [29] ‘nutrition support therapy’, [30] or ‘tube feeding’ [31]. Artificial nutrition can be delivered directly into the gut, enteral feeding; [28] or intravenously, parenteral feeding [32]. Common forms of artificial nutrition are: nasogastric (NG), in which a narrow plastic tube is passed through the nose into the stomach; and Percutaneous Endoscopic Gastrostomy (PEG), in which a tube is inserted directly into the stomach through the abdominal wall. Artificial hydration can be provided intravenously or subcutaneously [29].

#### Decisions about artificial nutrition and hydration

Decisions about artificial nutrition and hydration concern issues of life and death: weighing the potential for increasing morbidity and prolonging suffering, with potentially shortening life if no intervention takes place. Competing human rights must be measured and balanced, such as the right to life (European Convention on Human Rights (ECHR), Article 2), and the right to freedom from degrading treatment (ECHR, Article 3).^4^ The lack of a strong evidence base, particularly the dearth of Randomised Control Trials (RCT), contributes to the uncertainty in determining clinical outcomes [33]. For some interventions, such as PEG, treatment decisions can be particularly challenging. PEG insertions have been associated with: futile procedures, [34] significant mortality and morbidity rates, [34] and a lack of evidence of benefit in certain patient groups, such as those with advanced dementia [31]. These decisions are particularly difficult when someone is approaching the end of life, or when the individual lacks the mental capacity to be involved in the decision themselves. As with all medical treatments or interventions, the valid informed consent of the patient is central, but many patients who may benefit from artificial nutrition and hydration lack the decision-making capacity to consent to the procedure [35].

Our previous systematic literature review found that laws and healthcare practices concerning artificial nutrition vary internationally, but only found four studies which examined how decisions are made in practice [36]. There is a dearth of evidence concerning how decisions are made in real life clinical practice. This paper takes a principlist approach, reflecting upon the four classic principles of bioethical decision-making laid out by Beauchamp and Childress [37] to examine decision-making in practice within a UK hospital ‘Feeding Issues Multi-Professional Team’, in circumstances in which the ‘patient voice’ was limited, and in some cases absent (such as where the person was unconscious). These findings are then used to consider decision-making in national and international law. The key research questions are:

- How are decisions made concerning artificial nutrition for individuals at risk of lacking decision-making capacity?
- What are the key decision-making factors that are balanced?
- Who is involved in the decision-making process?

## Methods

### Service evaluation setting and methods

A weekly non-participant overt observation of a UK hospital Feeding Issues Multi-Professional Team (FIMPT) over three months of a year, and a one year retrospective study of the FIMPT records in 2011.

The FIMPT meets weekly to discuss patients with complex feeding difficulties, it aims to improve decision-making concerning assessment, treatment and clinical outcomes. The team comprises medical members from Gastroenterology, Palliative Care, Medicine for the Elderly, Speech and Language Therapy, Dietetics, Nutrition Clinical Nurse Specialists and Endoscopy Nurses. The team meets for around one hour to discuss patients referred each week. FIMPT meetings are part of normal clinical practice, they are not held as ‘best interests’ proceedings.

### Data collection and analysis

A thematic analysis approach was utilised for fieldwork, data analysis and interpretation [38]. For the first two months, detailed field notes on open topics were taken. For the last month, a subset of four meetings were sampled. These meetings were audio recorded and analysed alongside the detailed field notes. Notes were anonymised and the audio recordings were transcribed and anonymised. All notes and transcriptions were entered into NVivo 9 for analysis.

The thematic analysis was a six stage process: 1) Familiarisation with the data. The first author re-listened to the audio recordings and read and re-read the transcripts; 2) Initial coding stage. The transcripts were coded using both open inductive data driven coding and then coded using three broad categories derived from the research questions; 3) Searching for themes. Initial codes were discussed in team meetings between authors GC, SB and AH. Insights from theory were discussed and initial descriptive codes were developed into higher level analytical themes. 4) Reviewing the themes. Themes were reviewed and refined in an ongoing process of working with data in NVivo and discussing the findings in team meetings. 5) Defining and naming the themes. Themes were refined and reviewed by discussion. These were broadly separated into two over-arching themes; the decision-making process model and the interdependent decision-making axes, with 6 and 4 sub-themes respectively. 6) Producing the report. Final themes were written up to produce the paper which was reviewed and edited by all authors.

The retrospective analysis of records was undertaken using an electronic search of the hospital’s computer system for all FIMPT reports in 2011. Data was extracted into an anonymised form. Descriptive statistical analysis was undertaken using PASW Statistics 17.

### Ethics

After a review of the evaluation protocol by the committee chair of the NHS Research Ethics Committee (REC) Cambridgeshire 2, it was agreed on 08/03/2011 that the project was classed as a service evaluation and would not require further review by the REC. The researcher undertaking the fieldwork applied for, and subsequent to review, was awarded an honorary contract with the NHS Trust to undertake this work. The honorary contract bound the researcher to same standards of conduct and confidentiality as paid employees of the NHS Trust. This evaluation was carried out in compliance with the Helsinki Declaration [39].

### Anonymity, access and informed consent

To protect anonymity, the gender of all patients mentioned in this article has been redacted, and the year in which the observational study was undertaken has been removed. The observational study was not undertaken in the same year as the records study. Quotations have been edited to redact gender.

Access to the database utilised for the records study is not publicly available. It is only available to employees of the NHS Trust in which it was stored. The researcher accessing the database was bound by an honorary contract when she undertook the fieldwork. Data was extracted and anonymised on the NHS Trust premises.

After a review of the evaluation protocol, verbal consent was taken from the core members of the FIMPT team. After the analysis was completed, written informed consent was taken from the core members of the team for all anonymised quotations to be used in a publication.

## Results

### Patient characteristics

The one year study of FIMPT records revealed that in 2011, there were 206 patients referred using the hospital’s internal computer system. Of these, there were 158 separate patient cases and 48 re-referrals of a patient previously presented within the year. Ages ranged from 17 to 97 years, the mean age at referral was 65 years. It was recorded that, of the 158 patients, 85 (54 %) were judged to have decision-making capacity at all meetings, and 44 (30 %) lacked decision-making capacity or had uncertain decision-making capacity at one point in the decision-making process. The remaining 16 % had no statement about capacity in their record.

Most cases referred to the FIMPT were concerning an individual diagnosed with cancer. A primary diagnosis of cancer represented 70 individuals (44 % of all those referred) and 79 discussions held at meetings (38 % of all discussions). The second largest group to be referred were those who had been diagnosed with a Cerebral Vascular Accident (CVA). CVA patients represented 14 individuals (9 % of all those referred) and 24 discussions (12 % of all discussions) [35].

Over the one month sample of audio-recorded meetings in a different year, 12 patients were referred and 17 separate discussions were held. The largest group to be referred were those with a primary diagnosis of cancer (5 individuals, 42 % of all those referred). The remaining seven referred patients had primary diagnoses of: Cerebral Vascular Accident (CVA), Motor Neurone Disease (MND), Parkinson’s Disease, heart condition/disease, pneumonia, Down’s Syndrome with dementia, and unknown with dementia. Of this subset of 12 patients, half (50 %) had decision-making capacity throughout the entire decision-making process. Five patients (42 %) had no, or unclear decision-making capacity, at one point in the decision-making process; or had no/unclear capacity during the entire decision-making process. For one patient (8 %), no statement was made about their decision-making capacity.

For the analysis of the decision-making process all patient cases, both with and without decision-making capacity were analysed. For the analysis of factors involved in making decisions for those without decision-making capacity, the analysis focused upon only those cases involving patients with no or unclear decision-making capacity.

### The decision-making process

The discussions of patient cases followed a similar format at each meeting: 1) Forming the picture; 2) Identifying the problem; 3) Discussion; 4) Outcome and planning. (see Table 1).

**Table 1:** Cross-tabulation illustrating a model of the process of FIMPT decision-making based on a three month non-participant observation

Decision stage	Observation	Description
0. Before the meeting	Not observed	Patient referred from ward or community. Depending upon time of admission and time of referral: the dietician, speech and language therapist, gastroenterologist and other relevant specialists assess the patient. Decision-making capacity is assessed. Treatment options are talked over and explained with the patient and/or next of kin.
1. Forming the picture	Observed	Background information about the patient’s case is presented by member of the clinical team who knows the patient. Dietetics and speech & language therapy present the results from their assessments
2. Identifying the problem	Observed	If the reasons for the patient’s referral are apparent and their diagnosis is clear, the discussion can move straight onto stage 3. In complex cases, the Chair and other participants will ask further questions of the person presenting the case, the speech and language therapist, dietician and anyone else who has examined the patient.
3. Discussion	Observed	A deeper conversation about potential treatments and interventions. Conversation seeks to balance risks and benefits, other clinical issues, and includes ethical and social concerns. At this stage, the discussion has a less structured format. Stage 3 continues until the weight of evidence for a particular treatment option or course of action becomes apparent.
4. Outcome and planning	Observed	The Chair states the outcome of decision-making process and a brief discussion of treatment scheduling and planning follows. For some patients the outcome involves direct patient assessment by one of the FIMPT clinicians, this may include referral to palliative care or medicine for the elderly teams to assist in all future management not only management of nutrition.
5. After the meeting	Not observed	The recommendations from the meeting are presented back to the patient and/or next of kin by the treating team who have presented the patient at the meeting. Further discussions and decision-making take place. Relevant scheduling and planning takes place. The treating team return to the next meeting for further discussion if additional questions arise or if the patient’s condition changes.

Decision-making was not a singular decision about one method of artificial nutrition, but rather a process of problem-solving for patients who often had complex symptomatology and multiple co-morbidities. Decision-making was not just limited to team meetings. Patients were assessed on the ward before and after presentation at the FIMPT meeting, including an assessment of decision-making capacity. Discussions were ongoing, taking place both before and after the meetings. These discussions involved the ward team, relatives, and if possible, the patient themselves. This included review by FIMPT consultants, which may be required to assess technical aspects of tube placement, issues regarding decision-making capacity, discussions regarding patient place of care for outpatients, and on-going specialist palliative care input providing symptom control and advance care planning. For each individual patient, multiple treatment options and interventions were considered: ranging from continued observation, to changes in medication regimes, to artificial nutrition surgical interventions such as Percutaneous Endoscopic Gastrostomy (PEG).

### Balancing complex and multi-faceted decision-making factors

Decisions concerning artificial nutrition and hydration for patients at risk of lacking decision-making capacity were complex and multi-faceted. The topics of discussion varied for each individual case but the outcome of decision-making relied upon weighting and balancing the available information along four different but interdependent axes: (1) Risks, burdens and benefits; (2) Treatment goals; (3) Normative ethical values; (4) Interested parties. (see Table 2). These axes of decision-making are completely interdependent. For each decision, all available information has to be weighted or considered in relation to each axes which in turn have to all be considered and reconsidered in relation to one another.

**Table 2:** Cross-tabulation illustrating a model of the decision-making axes upon which clinical information was weighted to make decisions

Decision-making axes	Description
Risks, burdens and benefits	Comparison and weighting of the different treatment options and interventions by their potential effectiveness, dangers, outcomes and side-effects.
Treatment goals	The intended outcome, either specific to a particular treatment/interventions, place (institution/home) for future care for the patient or the overall intended outcome.
Normative ethical values	A balancing of actions in terms of ethical value. Actions in terms of their utilitarian value, i.e., increasing patient well-being and/or longevity; and deontological value, i.e., it was a ‘good’ course of action regardless of outcome.
Interested parties	Discussions incorporated the views of all involved stakeholders, which could include: the patient or their previous wishes, clinical team, relatives, etc.

#### (1) Risks, burdens and benefits

During the discussion of indicated treatments and interventions, the risks, burdens and benefits of each potential course of action were considered and balanced against one another. Different methods of feeding were compared to each other:

**Clinician 1:***I wonder what the benefit of a PEG over an NG would be for her/him?*

**Clinician 2:***It’s just caring for it at home, I think.*

**Discussion of a patient with Down’s Syndrome and dementia**

Where possible, the lowest risk approach with the least intervention was preferred:

**Clinician 1**: *Yes, it may be that with recovering from this infection s/he might even start feeding her/himself.*

**Clinician 2**: *Though s/he hadn’t last time really…*

**Discussion of an elderly patient with pneumonia and food refusal**

In the process of these ongoing discussions, the bioethical principles of non-maleficence and beneficence were utilised. Rather than conflicting with each other, the principles of non-maleficence and beneficence were utilised in a method of constant comparison, to continuously reassess and reflect upon the options. Considering different interventions and their associated risks, burdens and benefits was not a singular decision but rather an on-going process as the patient’s condition changed and dependent upon their circumstances. Acute issues, such as infections, were considered with the most urgency, but social and environmental factors were also taken into consideration. For example, the risks of treatments were considered both in terms of contraindications and in relation to the social environment the patient would be discharged to:

*I think it would also be worth getting another opinion from old-age psychiatry before s/he goes, to see if they’ve got any other suggestions about behaviour management, because it’s going to be very difficult for the family on the days that s/he refuses and they’re anxious at home. And if s/he gets another infection and won’t take any treatment, s/he’s going to end up back in here, isn’t s/he?*

**Discussion of an elderly patient with pneumonia and food refusal**

#### (2) Treatment goals

Risks and benefits were considered in relation to the overall treatment goals and intended health outcomes which could change as an individual’s condition developed and was reassessed. Treatment goals could be intervention specific, or broader relating to improvements in longevity and quality of life. These were not mutually exclusive goals. Rather, longevity and quality of life were both always important, but their weighting depended largely upon the individual patient’s prognosis. Although this in itself was not deterministic:

*Is s/he coming to the end of her/his life? You know, the bad days are getting worse and more frequent, s/he’s doing less well. It doesn’t necessarily mean s/he doesn’t… shouldn’t, have a PEG. But it’s the whole end of life planning, isn’t it? And the difficulty in feeding is just one aspect of it.*

**Discussion of a patient with Parkinson’s Disease**

For patients with treatable disease, or chronic long-term conditions, the focus was on extending life. Feeding interventions had to be aligned with disease altering treatments. Where it appeared that an individual may be coming to the end of their life, and the course of action was agreed with relatives and clinical team responsible, a greater emphasis was placed upon enhancing quality life.

**Clinician 1:***Would we be improving the quality or the quantity of her/his life by giving her a PEG?*

**Clinician 2:***Well, that’s a very difficult question…*

**Discussion of a patient with Down’s Syndrome and dementia**

Quality of life was conceptualised broadly and included balancing multiple clinical and non-clinical factors. Factors considered when assessing quality of life included weighing the ability to enjoy mealtimes and the taste of food with reducing the risk of aspiration:

*Is actually eating something giving her/him pleasure? …so the tastes, plus the risk of aspiration might be preferable?*

**Discussion of a patient with Down’s Syndrome and dementia**

**Clinician 1:** S/h*e’s just refusing all food at the moment.*

**Clinician 2:***Except on a good day, when s/he eats everything.*

**Clinician 1:***On Monday, s/he ate everything.*

**Clinician 2:***Is it the taste, do you think?*

**Clinician 1**: *Well, we’ve tried getting… we originally thought it was, so we got her/his family to bring in some really strongly-flavoured curries. S/he loves her/his Guinness and we prescribed Guinness, and on a good day, s/he’ll take that. S/he’ll happily drink that and you don’t even need to encourage her/him very much. But on a day when s/he doesn’t feel like doing anything, s/he just won’t, not even Guinness or her/his family’s encouragement will induce her/him to eat.*

**Discussion of an elderly patient with pneumonia and food refusal**

Quality of life was also seen to be enhanced by mealtimes with family, and spoon-feeding undertaken by relatives, was perceived as positive social interaction that could increase the individual’s quality of life. The impact of having a PEG fitted, and not being able to take food orally, was balanced against social interaction:

**Clinician 1:***What does the patient have to gain by feeding in a different way from how s/he’s been apparently successfully fed all this length of time at home, without the recurrent aspiration pneumonias?*

**Clinician 2:***The consultant yesterday said that he felt s/he still had some quality of life in the interactions that s/he shares with her/his family. And I think they were suggesting PEG as an option to kind of try and avoid the aspirations that might possibly occur…*

**Clinician 1:***It doesn’t necessarily though, does it?*

**Clinician 2:***No. Exactly*.

**Clinician 1:***Especially if s/he’s lying flat on her/his back… And your description it sounds as if the interaction s/he has with her/his family is when they sit her/him up, wake her/him up to feed he/him*

**Discussion of a patient with Down’s Syndrome and dementia**

**Clinician 1:***But at the moment s/he doesn’t really have any interaction with her/his family even?*

**Clinician 2:***Well, s/he talks to her/his family.*

**Clinician 1:***And s/he will, s/he’ll drink with them, s/he refuses most other times but they’re trying to get her/him to eat but so far s/he’s refused.*

**Discussion of an elderly patient with pneumonia and food refusal**

A stimulating and interesting environment was also considered important, for both inpatients, and after being discharged:

*S/he’d come from a side room because of her/his C Diff and they’re hoping that s/he’ll find the bay a bit more stimulating and s/he’ll get on a bit better, so I think what they were hoping was to give her/him one more try with an NG with antidepressants.*

**Discussion of an elderly patient with dementia**

*Before admission… s/he had a pretty good quality of life, s/he lives with her/his extended, very supportive family who are keen that s/he stays at home and wouldn’t want nursing home influence, so it was really just a question of how we could best manage her/him.*

**Discussion of an elderly patient with pneumonia and food refusal**

A central quality of life factor was freedom from pain and discomfort. Family members particularly voiced this concern for their relative. Although family members did not attend FIMPT meetings, family views were sought by the treating team, and raised at the meetings for patient’s lacking capacity:

*S/he does have family, they’ve been a bit concerned, and originally they were sort of reluctant about an NG. They had the impression that it was cruel, and not the right thing to do.*

**Discussion of an elderly patient with dementia**

#### (3)Normative ethical values

Any treatment or course of action was balanced in terms of its normative ethical values. Treatment options were balanced between those that had a utilitarian value, in that they increased patient well-being, prevented harm or helped a course of treatment proceed; and those actions that had a deontological value, in that they were a ‘good’ in themselves regardless of outcome. Decision-making was easier in the cases in which the normative values aligned. For example, when making the decision to spoon feed a patient:

**Clinician 1:***And have we actually had a try of sitting there with a teaspoon?*

**Clinician 2:***I think that’s what the hope is that when s/he’s on* [Ward name] *perhaps a little bit more time to do that.*

**Discussion of an elderly patient with dementia**

In this scenario, food as nourishment to improve the patient’s health and physical condition align with food and social contact through spoon-feeding as pleasure and enjoyment to maximise the patient’s wellbeing. However, when the value of food as pleasure conflicts with the aims of improving the patient’s health and well-being, it is clear that decisions are not just being made to maximise overall utilitarian values. For example, when inserting a PEG feeding tube was considered:

**Clinician 1**: *Yeah, the husband/wife did also mention about the medication that it would be easier for them to administer medication through the PEG…*

**Clinician 2**: *So it would take a bit of the burden off them worrying about it.*

**Clinician 1:** …*but they might not actually use it completely for nutrition but initially to supplement it, um, but actually use it basically for medications, that was also brought up by her/his husband/wife.*

**Discussion of a patient with Parkinson’s Disease**

In this scenario, despite the risk of choking, some oral feeding will still take place. Food as pleasure and social contact through oral feeding have become valued as intrinsic goods within themselves, regardless of the potential consequences i.e., that oral feeding could ultimately lead to choking and the patient’s early death. Decisions are no longer just about maximising overall utility, they are made more difficult because of the conflict between the utilitarian values of preventing aspiration and administering medicine with ease, and the intrinsic good of being able to enjoy food and family mealtimes

#### (4) Interested parties

The meetings were not the sole locus of decision-making. Decisions also involved: the clinical team treating the patient; relatives, often spouses or children; and if possible, the patient’s previous or interpreted wishes. Discussions held at the FIMPT had to incorporate the views of all these interested parties. The treating clinical team were usually represented at the meetings, but family members were not. Instead, in-depth conversations were sought before and after. Communication with family was two-way; relatives’ views on treatment decisions were sought and clinicians would feedback about potential interventions:

*Yeah. I mean, I can certainly talk to the family and give them a call or hopefully they’ll be in next time I review the patient and go through those options in more detail.*

**Discussion of a patient with Down’s Syndrome and dementia**

*I know there were discussions, it was raised in the last meeting about the possibility of a PEG and I think, from her/his family’s point of view, I think this is something that they would like, because they obviously want her/him to be at home, they want her/him to be out of hospital and, from her/his point of view, it’s tricky, because as with most of his care, sometimes s/he’ll say “Yes, that’s fine, I want to do that” and sometimes s/he will refuse everything*.

**Discussion of an elderly patient with pneumonia and food refusal**

Sometimes this involved managing relatives’ expectations about what could be achieved through artificial feeding interventions:

**Clinician 2:***Her/his BMIs?*

**Clinician 1:***Twenty-six, so…*

**Clinician 2:***Yeah, it’s fine. So, s/he’s not malnourished, so we’d just be maintaining her/him whilst her/his disease got worse. So it’s making sure they understand that.*

**Discussion of a patient with Parkinson’s Disease**

Family interactions were also important in terms of care planning, particularly if the patient lived with family members. Though the living situation of a patient did not dictate whether a form of artificial nutrition was recommended or not, family relationships were discussed as part of the decision-making process. Where appropriate the patient was not just treated as an individual but also as part of a family unit.

*We just need to train and just need to find out the situation with her/his family actually, you know, in terms of… the reality is, how much they actually want to take on and in terms of there would need to be a care package put together and we’d need to get the carers trained, so I think, yeah.*

**Discussion of an elderly patient with pneumonia and food refusal**

Patient’s own wishes could be incorporated into the decision-making if they were available. However, none of the individuals referred to FIMPT during the three month period had made Advance Decisions to Refuse Treatment (ADRT) related to feeding interventions. One individual with decision-making capacity and Motor Neurone Disease, had made an advanced expression of treatment preference regarding Percutaneous Endoscopic Gastrostomy.

Patient’s informal wishes about interventions were sought through relatives, where they were available:

*I think we need to know what her/his family think, if the family know what her/his views would be, not that that determines what we do, but we need to take that into account, if there’s any way of understanding if s/he can swallow or not but I think probably we wouldn’t be able to do that because s/he’s drowsy…*

**Discussion of an elderly patient with dementia**

In the majority of cases, no formal or informal information was available about what a patient might want. In these cases, where appropriate, an interpretation of the patient’s behaviour was considered. For example, in the case of an elderly patient with dementia who has stopped eating and drinking, the meaning of this behaviour is discussed:

*Is it priority nutrition in this case? Is it the end of her/his….coming to the end of her/his life that’s part of it? Or as part of dementia? And if it’s a case of we think s/he’s really depressed and needs antidepressants then tube feeding is probably inappropriate?*

**Discussion of an elderly patient with dementia**

In another case, the meaning of an elderly patient refusing food and water is considered:

*I think her/his family felt that it was more of a fear of choking or aspirating that was preventing her/him because that’s what s/he told before s/he was ill, that s/he was scared, I don’t know whether that’s contributing to her/his food refusal still.*

**Discussion of an elderly patient with pneumonia and food refusal**

Through these kinds of discussions, the bioethical principle ‘respect for autonomy’ was realised, despite the patient not being fully autonomous, or having decision-making capacity themselves.

## Discussion

This service evaluation of a Feeding Issues Multi-Professional Team (FIMPT) based within a UK hospital has revealed that decision-making concerning feeding interventions for individuals who lack capacity is a dynamic and complex process. The decision-making was not a one-off choice, but rather involved many different steps and many decisions. Discussions involving relatives and other clinicians, were also part of the process, taking place both before and after team meetings. The decision-making process evolved as the patient’s condition changed; sometimes involving re-referring and re-discussion at FIMPT meetings.

Rather than a specific set of decision-making factors or criteria which can be applied to each case, decision-making is better conceptualised as process of weighing and balancing information on the inter-related axes of: (1) Risks, burdens and benefits; (2) Treatment goals; (3) Normative ethical actions; (4) Interested parties. However, there were some key issues discussed across many of the cases: acute issues were given the greatest weight, but wider social and environmental were also taken into account; and quality and length of life were always both considered. Key quality of life factors included pleasure of food versus risk of aspiration, social contact time versus ease of feeding and medication administration; freedom from pain and discomfort versus not prolonging suffering.

When individuals lack decision-making capacity and are unable to give informed consent, the usual ethical processes of medical procedures are disrupted. However, even though the individuals were not fully autonomous, within this example of decision-making, the principle of ‘respect for autonomy’ was still partially realised as patients’ behaviour was taken into account, and clinicians spoke to patients’ relatives. As ‘respect for autonomy’ could not be fully utilised, the principles of non-maleficence and beneficence gained greater significance. Non-maleficence and beneficence were utilised through constant comparison in verbal group reasoning, allowing discussions about treatment goals, risks, burdens and benefits. The fourth bioethical principle of ‘justice’ was not utilised within the discussions, as each case was considered on its own terms, and not in relation to other patients or to resource management. Balancing the normative ethical values made decision-making easier. When a utilitarian course of action, also had deontological value, less debate was required about the course of action.

The observational study revealed a complex and often ‘messy’ reality of decision-making in the real world; decisions were always ongoing, involving multiple recommendations and decision-makers. The findings from this study reveal there is no clear distinction between supported and substituted decision-making, because in real world clinical practice decisions do not exist in isolation: decisions involved multiple stages; some of which involved substituted decisions, such as deciding which treatments to recommend; and some of which involved supported decision-making such as taking into account a patient’s behaviours, even if they didn’t have full decision-making capacity. Perhaps the distinction between substituted and supported decision-making could be better conceptualised as a sliding scale, in which parts of decisions requiring substitution decisions should always be in a patient’s ‘best interests’, but capacity should also be maximised for supporting decisions at the same time. The recent report by the House of Lords on the Mental Capacity Act, criticised the lack of implementation [40]. The findings from this observational study show the complexity of real world clinical decision-making. This may go some way to explaining the difficulties of implementation of the MCA. The findings also reveal the importance and robustness of multi-professional discussions in these settings, the same level of debate could not be achieved by a uni-professional discussion. This paper reports upon a small scale service evaluation. Further research could examine these models of decision-making in other clinical settings.

## Conclusions

Decisions regarding eating and drinking interventions at the end of life are serious. In particular, decisions surrounding stopping oral feeding and inserting feeding tubes, The decision to intervene, or not intervene, with feeding is ethically and clinically challenging, and often occurs under grave circumstances. Team meetings addressed these decisions using a formalised process operating within the framework of the Mental Capacity Act 2005, which requires decision-makers to act according to the patient’s best interests. The perspectives of different disciplines concerning what might be the most appropriate course of action were encouraged and sought during discussions.

Decision-making regarding feeding interventions involves multiple decision-makers and decision points, and it is acknowledged that the study has not addressed all of these, or seen the entire picture. The ward consultant responsible for the patient’s care was not usually present at the meeting, often the case was present by a junior medical member of their team. The study did not address how decision-making capacity had been assessed by the ward team, or how it was reviewed by the FIMPT, or information was relayed to patients and their families. For decisions concerning the insertion of percutaneous endoscopic gastrostomy (PEG), the consultant who would potentially insert the PEG was present. However, the study did not include subsequent consultations with patient and family after the team meeting to explain the decision to proceed with an intervention and obtain informed consent or assent. The multiple points of decision-making, and the number of people involved with the decision-making process, mean the question of ‘who decides’ cannot be fully answered. There is a potential for anonymity of multiple decision-makers to arise. While the meetings operate within the Mental Capacity Act 2005 (MCA), what the paper describes does not constitute a ‘best interests proceeding’ under the MCA, instead it is part of routine clinical practice. The team did not assess decision-making capacity at meetings, but did review the assessments. Patient’s relatives did not attend the meetings, but rather their views were sought. The team were one-step removed from the patient’s bedside, and thus unable to make best interests decisions. It could be reasoned that the team meetings made healthcare or “medical best interest” decisions, deciding on the right medical treatment while using MCA ‘best interests’ language.

Some legal interpretations of the United Nations Convention on the Rights of Persons with Disabilities (UNCRPD), argue that under the UNCRPD there is no point at which legal decision-making capacity is lost, and thus all decisions must be supported, rather than substituted [18–20]. Under the Mental Capacity Act 2005, legal capacity testing represents a ‘cut-off point’ for a decision, and creates a categorical distinction between supported and substituted decision-making. This study has illustrated that, in clinical practice, decisions may contain elements of both substituted and supported decision-making, often moving backwards and forwards between the two. Substituted and supported decision-making may be better represented as existing on a continuum.

## Footnotes

1 Department of Health. *Mental Capacity Act*. London: Stationary Office, 2005.

2 Scottish Executive. *Adults with Incapacity (Scotland) Act*. Edinburgh, Scottish Executive, 2000.

3 United Nations. Convention on the Rights of Persons with Disabilities and Optional Protocol. UN, 2006.

4 Council of Europe, *European Convention for the Protection of Human Rights and Fundamental Freedoms, as amended by Protocols Nos. 11 and 14*, 4 November 1950
